# Peak steps to measure ‘‘capacity for activity’’: Actigraphy in the ADAPT registry with oral treprostinil

**DOI:** 10.1016/j.jhlto.2025.100219

**Published:** 2025-01-23

**Authors:** Daniel Lachant, James Gagermeier, Scott Seaman, Andrew Wang, Meredith Broderick, R. James White

**Affiliations:** a Pulmonary & Critical Care Medicine, University of Rochester Medical Center, Rochester, New York; b Department of Medicine, Loyola University Medical Center, Maywood, Illinois; c United Therapeutics Corporation, Research Triangle Park, North Carolina

**Keywords:** pulmonary arterial hypertension, PAH, movement, actigraphy, oral treprostinil, steps

## Abstract

Pulmonary arterial hypertension (PAH) patients have low activity time. Actigraphy provides an objective quantification of movement outside of clinic visits (home, work, social activities). Summary actigraphy measures (total daily steps or activity time) have not been shown to increase after adding therapies in patients with PAH. We developed a novel treatment responsive metric, Peak Steps, to measure the ‘capacity for movement’ in patients with PAH. Using data from the ADAPT registry (NCT03045029), we calculated Peak Steps and used summary actigraphy measurements and correlated it with six-minute walk distance (6MWD) and emPHasis-10 quality of life scores. After exposure to oral treprostinil, we observed a significant increase in Peak Steps, but no change in total daily steps or activity time. Peak Steps correlated strongly with 6MWD and emPHasis-10 scores. We propose that Peak Steps is a more sensitive marker of treatment response than traditional actigraphy parameters, which can have large behavioral influences.

As pulmonary arterial hypertension (PAH) progresses, patients alter their daily routine leading to a decrease in daily activity.^1^ Actigraphy is an objective way to quantify this movement in the home setting and provides complementary information to clinic assessments. It is unclear if activity is consistently related to right heart function.^2^, ^3^ Traditional summary actigraphy metrics were not therapy responsive in a selexipag clinical trial.^4^ We developed “Peak Steps”, a novel metric of ‘‘capacity for activity’’ to detect improvement using actigraphy after intensifying therapy and after a behavioral intervention.^5^ We have not previously tested it with multi-center data.

Using the ADAPT Registry, the aim of this post-hoc study was to evaluate whether Peak Steps and traditional activity metrics (measured by wrist-worn ActiGraph) change after open-label oral treprostinil treatment. We also correlated the activity parameters with emPHasis-10 and six-minute walk distance (6MWD). We hypothesized Peak Steps would be a better marker of treatment response than total activity parameters because the latter are heavily influenced by behavior and daily routine.^1^

## Methods

ADAPT (NCT03045029) was a prospective, multicenter observational registry of patients with Group 1 pulmonary hypertension (PAH) started on oral treprostinil (within 182 days). The institutional review board or independent ethics committee approved the protocol for each site.

There was an optional actigraphy sub-study; all data are from the sub-study and 2 associated standard of care visits (Visit 1 and Visit 2). Visit 1 was not necessarily the baseline visit of ADAPT; patients could have enrolled in the actigraphy sub-study after entry into ADAPT. Visit 2 was the next consecutive clinic visit. Actigraphy data were collected using an ActiGraph CentrePoint Insight Watch (Pensacola, FL) and were analyzed only for participants with data at both clinical visits. Per the protocol, patients were instructed to wear the watch for 10 consecutive days during waking hours immediately following Visits 1 and 2. However, due to non-compliance with wearing the watch, we included patients in this post-hoc analysis that wore the ActiGraph watch for >10 hours of wear time per day on at least 3 days following Visits 1 and 2. At Visit 1 in the sub-study, participants may or may not have been taking oral treprostinil. Visit 2 was the next standard of care visit and occurred between 2 and 12 months after Visit 1. N-terminal prohormone of brain natriuretic peptide (NT-proBNP), 6MWD, functional class, and emPHasis-10 were also collected during standard of care visits.^6^

Traditional metrics included total daily steps, light activity, and moderate activity time (defined by the ActiGraph algorithm). Peak Steps were calculated as previously described.^5^ Briefly, we binned daily steps into 1-minute epochs and rank-ordered the epochs of each day (contiguous or not) by the highest number of steps. The epochs with the most steps were the ‘‘Peak Steps’’ (e.g., ‘‘Peak 5 minutes’’ = total steps in the top 5 of the day’s 1 minute intervals).^5^ ‘‘Peak Steps’’ were then averaged (mean) for the days worn immediately after each study visit.

The two-tailed Wilcoxon matched-pairs signed rank test was used to compare Visits 1 and 2; Spearman (non-parametric) coefficients were used to correlate 6MWD and emPHasis-10 quality of life data with activity data. GraphPad Prism 9 was used for analysis.

## Results

One hundred eighty-one participants enrolled in ADAPT with 22 of those enrolling in the optional actigraphy sub study. Thirteen participants from 8 US sites had paired data with sufficient wear time during both monitoring periods between 2019 and 2021; none of these participants reported side effects related to wearing the CentrePoint Insight Watch. Eight of these 13 patients were already on oral treprostinil at Visit 1 (‘‘Oral Treprostinil Exposed Group’’) and 5 were not on oral treprostinil at Visit 1 (‘‘Oral Treprostinil Naïve Group’’). In the ‘‘Oral Treprostinil Exposed Group’’, 3 patients increased their oral treprostinil dose, 4 maintained their oral treprostinil dose, and 1 decreased their oral treprostinil dose from Visit 1 to Visit 2. Nine participants had a single wear period and were not included in this analysis. Demographics at entry of ADAPT are provided in Table 1.

**Table 1:** Demographics at Entry of the ADAPT Registry

	N = 13
Age, years, median (IQR)	51 (39, 68)
Female Sex, N (%)	10 (76.9)
BMI, kg/m^2^, median (IQR)	27 (25, 34)
PAH Type, N (%)	
Idiopathic or Heritable	9 (69)
Associated with connective tissue disease	3 (23)
Associated with congenital heart defect	1 (8)
Background PAH Therapies, N (%)	
None	1 (8)
Monotherapy	5 (38)
Combination therapy (ERA + PDE5i or sGC)	7 (54)
Visit 1 Oral Treprostinil Dose, mg every 8 hours, median (IQR), Oral Treprostinil Exposed Group (N = 8)^a^	4.5 (1.7, 5.4)
Visit 2 Oral Treprostinil Dose, mg every 8 hours, median (IQR), Overall Cohort (N = 13)	5.0 (3.0, 7.6)
Visit 2 Oral Treprostinil Dose, mg every 8 hours, median (IQR), Oral Treprostinil Exposed Group (N = 8)	5.0 (3.8, 5.5)
Visit 2 Oral Treprostinil Dose, mg every 8 hours, median (IQR), Oral Treprostinil Naïve Group (N = 5)	3.4 (3.0, 5.0)
Time Between Visits, months, median (IQR)	6 (3, 8)
WHO Functional Class, N (%)	
I	1 (8)
II	8 (62)
III	4 (31)
6MWD, meters, median (IQR)	378 (306, 463)
BNP, pg/ml, median (IQR) (N = 7) NT-proBNP, pg/ml, median (IQR) (N = 5)	20 (20, 208) 159 (90, 746)

6MWD, six-minute walk distance; BMI, body mass index; BNP, brain natriuretic peptide; IQR, interquartile range; NT-proBNP, N-terminal prohormone of brain natriuretic peptide; PAH, pulmonary arterial hypertension.

a Eight subjects were taking oral treprostinil at baseline for a median of 4 months (Oral Treprostinil Exposed Group). Five subjects were not taking oral treprostinil at baseline (Oral Treprostinil Naïve Group).

**Figure 1:**
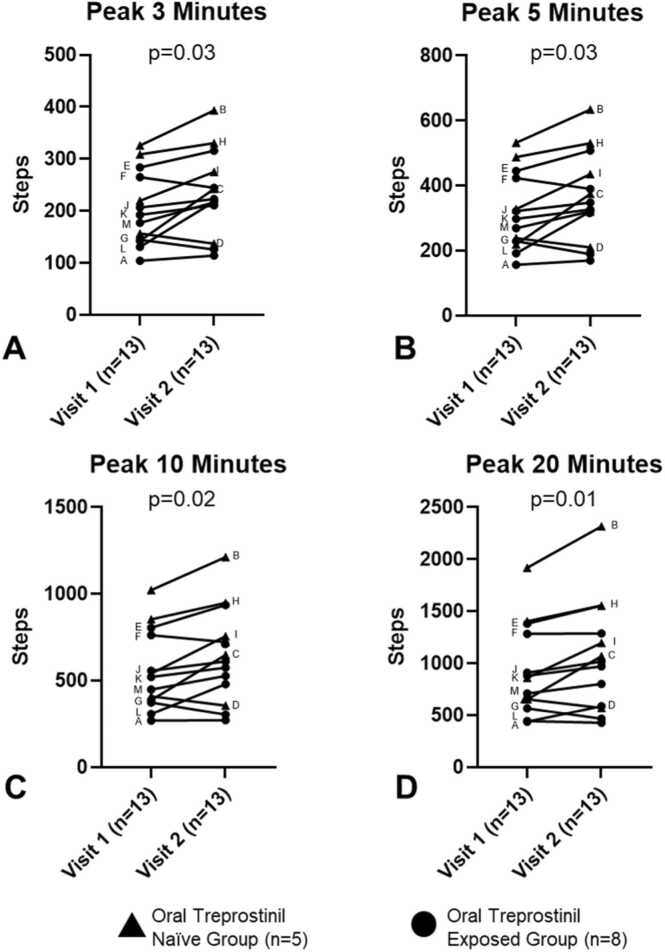
Changes in peak steps between Visit 1 and 2. (A–D) Peak 3–20 minutes steps increased with oral treprostinil treatment. The Oral Treprostinil Naïve Group (*n* = 5) is denoted by triangles and the Oral Treprostinil Exposed Group (*n* = 8) is denoted by circles. Each of the 13 participants is denoted by a letter. Peak steps are a mean over the days worn immediately after each study visit.

**Figure 2:**
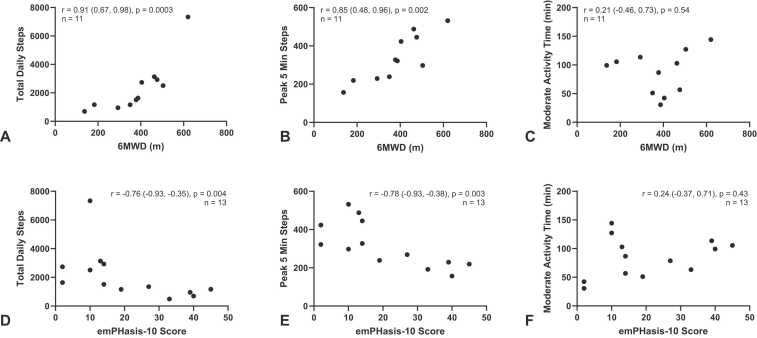
Six-minute walk distance (6MWD) and emPHasis-10 correlate with total daily steps and 5-minute peak steps at Visit 1. (A,B) Both baseline total daily steps and peak 5-minute steps have a strong positive correlation with 6MWD at Visit 1. (C) Moderate activity did not correlate with 6MWD at visit 1. (D,E) Total daily steps and peak 5-minute steps have a strong negative correlation with emPHasis-10 at Visit 1. (F) Moderate activity time did not correlate with emPHasis-10 at Visit 1. Total daily steps, peak 5-minute steps, and moderate activity time are a mean over the days worn immediately after the baseline study visit.

There was no difference in median wear time between Visit 1 and 2, (861 [767, 1,228] vs 991 [853, 1,119] minutes, *p* = 0.9). Between Visit 1 and 2, there was no change in median daily steps, (1,512 [1,166, 2,738] vs 2,296 [817, 2,631] steps/day, *p* = 0.5; median light activity, 213 [172, 246] vs 199 [168, 213] minutes, *p* = 0.9; or median moderate activity, 87 [57, 106] vs 87 [36, 119] minutes, *p* = 0.9). Peak Steps (3, 5, 10, and 20-minute intervals) increased between Visit 1 and Visit 2 (Figure 1). Both daily steps and Peak Steps positively correlated with 6MWD and negatively correlated with emPHasis-10 as expected (lower emPHasis-10 scores indicate better patient-reported health) (Figure 2). Light and moderate activity time did not correlate with 6MWD or emPHasis-10. Six-minute walk distance negatively correlated with emPHasis-10 (higher emPHasis-10 scores indicate poorer health). Between Visit 1 and Visit 2, there was no significant difference for 6MWD (*p* = 0.97) or emPHasis-10 (*p* = 0.73) using the two-tailed Wilcoxon matched-pairs signed rank test.

## Discussion

In this multicenter observational study, “Peak Steps” increased between Visit 1 and Visit 2, while daily steps and activity time remained unchanged. We and others have proposed that traditional actigraphy metrics are influenced by many factors (e.g., intrinsic behavior, weather, and medication side effects) beyond PAH; thus, making it difficult to detect therapy-associated changes (changes presumably attributable to physiologic improvement). Change in peak steps may be a better measure of capacity for activity than traditional actigraphy measurements because it measures brief, higher intensity activity when an individual intends/desires to move and, because of therapy, is better able to do so.

Given the low activity time in the presented and historical data, we initially expected increased activity time after adding oral treprostinil.^1^ However, we observed no increase, which is consistent with other data in PAH^4^, ^5^ and interstitial lung disease.^7^, ^8^ For this and other serious chronic illness, patients may have curtailed their daily routines to accommodate their physical limitations so that any therapy-associated improvement does not translate into an increase in total activity. Additionally, long standing deconditioning, depression, and anxiety may also mute any increase in daily activities. Emphasizing the behavioral aspect of activity, a behavioral intervention program delivered to participants' mobile devices increased daily steps in stable patients with PAH, highlighting that daily activity metrics measure more than physiologic severity.^9^

Furthermore, algorithms developed for healthy individuals may be inappropriate for chronically ill patients with altered stamina and gait, especially those needing supplemental oxygen.^10^ One-minute of activity is credited when a certain degree of acceleration is reached; the algorithms employed use healthy controls and then characterize that one-minute of activity as light or moderate.^10^ The ‘‘moderate’’ activity threshold for healthy individuals is at the outer limit for a majority of PAH patients (for context, see Lachant and White^1^); it is not surprising that therapy did not increase total activity time measured by algorithms tuned for healthy people. In contrast, Peak Steps (the top one-minute intervals) analyzes granular data to show a therapy-associated improvement in brief, desired ‘‘bursts’’ even when total activity is more constant. In this open-label registry, Peak Steps also correlated with 6MWD and emPHasis-10. Peak Steps were treatment-responsive in that it showed increased walking speed (movement) in one minute intervals in the home setting after being on oral treprostinil. Although we lacked randomization and a control group, the Hawthorne effect reduces the likelihood of observing increased activity; participants generally increase activity while being monitored during the first observation period, but relax during the second. In 2 previously published cohorts, the control groups did not increase Peak Steps during follow-up assessment, so it seems less likely that this was a chance finding.^5^, ^9^

We are very limited in the conclusions we can draw from this retrospective analysis of an open-label study without a control group. The cohort of patients analyzed in this study is very small and, as such, conclusions should be tempered. Paired follow-up was greatly limited by the COVID-19 pandemic, which also influenced activity and was likely the reason why moderate activity did not correlate with 6MWD or emPHasis-10. Changes in activity attributable to therapy could have been muted, as 8 patients had been taking oral treprostinil for up to 182 days before Baseline. We used a wear time threshold of 10 hours to include data analysis and may have missed some activity. This is higher than other reports that used 5 hours.^2^ Due to the small heterogenous sample, there was not enough power to compare changes in 6MWD and emPHasis-10 with changes in actigraphy.

In conclusion, Peak Steps in a multi-center study may be a more sensitive measure to change after adding therapy than daily steps or arbitrary activity thresholds. The correlation with emPHasis-10 suggests that Peak Steps measures activity that matters to patients. Peak steps could allow remote monitoring for therapy response in clinical practice and research. Peak Steps and cadence are being applied to other cardiopulmonary conditions such as chronic obstructive pulmonary disease^11^ and pulmonary embolism.^12^ Further controlled studies are warranted to better understand the relationship between changes in physiology and PAH outcomes interest with different actigraphy parameters.

## Author contributions

DL actively recruited and treated patients in the study and served as the lead for data analyses and interpretation. JG actively recruited and treated patients in the study and aided in data interpretation. RJW actively recruited and treated patients and aided in data interpretation. DL, RJW, and SS wrote the initial draft of the manuscript. MB and AW aided in data analyses and interpretation. All authors participated in revising the drafts of the manuscript. All authors reviewed the final version of the manuscript and approved the decision to submit the manuscript.

## Conflicts of interest statement

The authors declare the following financial interests/personal relationships which may be considered as potential competing interests: All authors (Daniel Lachant, James Gagermeier, Scott Seaman, Andrew Wang, Meredith Broderick, R. James White) reports financial support, administrative support, article publishing charges, equipment, drugs, or supplies, statistical analysis, and writing assistance were provided by United Therapeutics Corporation. Daniel Lachant reports a relationship with United Therapeutics Corporation that includes: Consulting or advisory and speaking and lecture fees. James Gagermeier reports a relationship with United Therapeutics Corporation that includes: Consulting or advisory and travel reimbursement. Scott Seaman reports a relationship with United Therapeutics Corporation that includes: Employment and equity or stocks. Andrew Wang reports a relationship with United Therapeutics Corporation that includes: Employment and equity or stocks. Meredith Broderick reports a relationship with United Therapeutics Corporation that includes: Employment and equity or stocks. R. James White (all funds to institution) reports a relationship with United Therapeutics Corporation that includes: Consulting or advisory. R. James White (all funds to institution) reports a relationship with Merck that includes: Consulting or advisory. If there are other authors, they declare that they have no known competing financial interests or personal relationships that could have appeared to influence the work reported in this paper.
